# Eating behaviors in the digital age: the role of social media and healthy diet literacy

**DOI:** 10.1007/s40519-025-01808-2

**Published:** 2026-01-30

**Authors:** Muhammet Ali Çakır, Selma Fırat

**Affiliations:** Department of Nutrition and Dietetics, Faculty of Health Sciences, Kırklareli University, Kırklareli, Turkey

**Keywords:** Social media addiction, Eating behavior, Digital diet literacy, Healthy nutrition, Adults

## Abstract

**Purpose:**

The growing use of social media has increased access to online health and nutrition information, which may influence individuals’ eating behaviors. This study examined the relationship between digital healthy diet literacy, social media addiction, and eating behaviors among adults.

**Methods:**

This cross-sectional study included 554 adults aged 18–64 years in Kırklareli, Türkiye. Data were collected through face-to-face interviews using a Personal Information Form, the Social Media Addiction Scale (SESMEB), the Bergen Social Media Addiction Scale (BSMAS), and the Digital Healthy Diet Literacy Scale (DHDL). Anthropometric measurements were obtained by the researchers.

**Results:**

The participants’ mean age was 31.5 ± 10.4 years; 52% were female and 43.5% university graduates. SESMEB and BSMAS scores were higher among younger, single, and more educated individuals but lower among obese participants (*p* < 0.05). DHDL scores were higher in those with higher education, regular exercise, and sufficient income, but lower in obese participants (*p* < 0.05). Significant relationships were found between all scales and social media use characteristics (number of accounts, duration, importance of likes, participation in nutrition groups, and exposure to advertisements). Strong positive correlations were observed among SESMEB, BSMAS, and DHDL scores.

**Conclusions:**

Social media addiction and digital healthy diet literacy are inter-related and may influence eating behaviors. Enhancing digital health and nutrition literacy may help reduce negative effects of social media and promote healthier eating habits.

*Level of evidence*: Level III, cross-sectional analytic study.

## Introduction

The widespread use of the Internet and the advancement of smartphone and computer technologies have significantly facilitated individuals’ access to health-related information [1]. These developments have enabled people to utilize online resources to seek health advice, particularly about diet, weight control, and disease prevention, learn about diseases, and review medical opinions. Social media platforms have played a pivotal role in this digital transformation by serving as central spaces for information exchange and the creation of online communities [2]. However, the difficulty in determining the reliability and scientific quality of health information obtained online poses potential risks that may negatively affect health behaviors [3].

Excessive and uncontrolled use of social media, when it reaches an addictive level, has been associated with various health problems, such as body dissatisfaction, low self-esteem, depression, and disordered eating behaviors [4, 5]. In particular, exposure to idealized body images can lead individuals to compare their own bodies and experience dissatisfaction [6]. This phenomenon can have serious psychological and physical consequences not only among adolescents but also among adults. Furthermore, the frequent sharing of food-related content and advertisements for unhealthy foods on social media platforms can adversely influence individuals’ eating behaviors [7–13]. The literature indicates strong associations between social media use and unhealthy eating habits [14]. Disordered eating behaviors increase the risk of developing eating disorders and can result in serious medical and psychological outcomes [15]. Therefore, understanding the factors contributing to negative eating behaviors, particularly among adults, and developing strategies to prevent them is of critical importance.

On the other hand, social media also presents positive opportunities in addition to its adverse effects. Health professionals and public institutions can employ social media as a tool to facilitate access to accurate information, promote healthy lifestyles, and raise awareness among individuals [16]. Nevertheless, the abundance of unregulated and misleading content may lead individuals toward unscientific diet practices and poor nutritional habits. Whether individuals benefit from these opportunities or are harmed by misleading content likely depends on their ability to locate, critically appraise, and apply nutrition information obtained online. Hence, it is important to evaluate both the positive and negative impacts of social media on eating behaviors.

In recent years, one concept that has gained prominence in this context is Digital Healthy Diet Literacy (DHDL). DHDL refers to individuals’ ability to find, evaluate, and apply information related to healthy nutrition in online environments [17]. Studies have shown that higher levels of DHDL are associated with healthier eating habits, better mental health, and improved quality of life [1, 17, 18]. Moreover, individuals with higher digital literacy levels are reported to evaluate social media content more critically, thereby reducing their risk of developing addictive behaviors [19].

However, studies that simultaneously examine the connections between social media addiction, digital healthy diet literacy, and eating behaviors are still limited. While earlier research has linked social media use to body image issues and disordered eating, only a few studies have explored digital healthy diet literacy as a possible protective factor in the relationship between social media addiction and social media-related eating behaviors. This cross-sectional study aims to fill this gap by investigating the combined and potential moderating effects of social media addiction and digital healthy diet literacy on social media-related eating behaviors in adults. We hypothesized that higher social media addiction would be associated with more unhealthy eating behaviors tied to social media, that higher digital healthy diet literacy would be associated with healthier eating habits, and that increased literacy would weaken the association between social media addiction and these behaviors. In addition, we examined how demographic factors, eating habits, and patterns of social media use were related to these variables.

## Methods

### Study design and participants

**Fig. 1:**
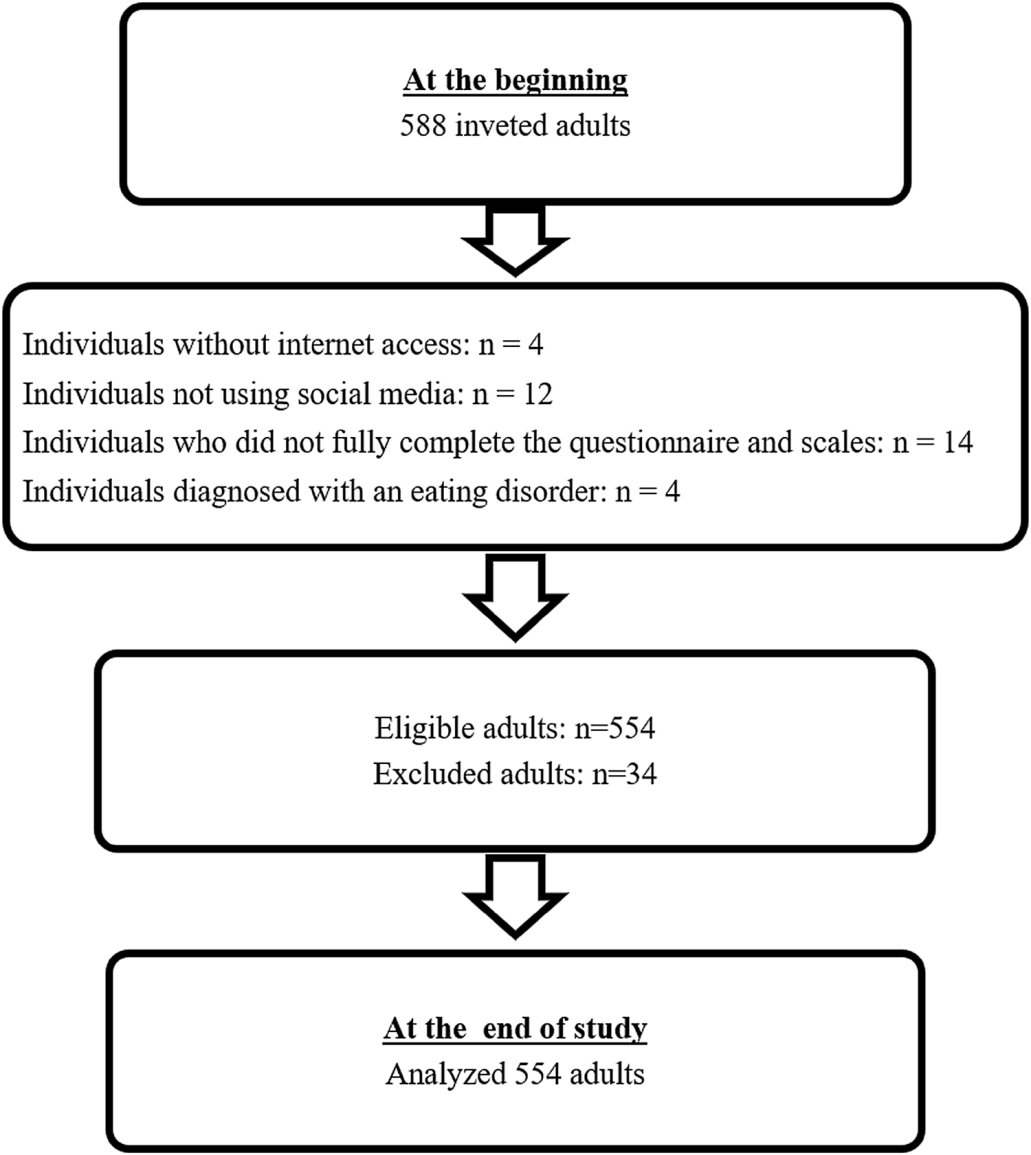
Study flow diagram

This cross-sectional study was conducted among community-dwelling adults residing in Kırklareli, Türkiye, using a community-based convenience sample. According to data from the Turkish Statistical Institute (TÜİK, 2023), the total population of Kırklareli in 2023 was reported as 377.156. The study population consisted of individuals aged 18–64 years (*n* = 246.256). The sample size was calculated using the G*Power 3.1.9.4 program, with a significance level of *α* = 0.05, a statistical power of 85% (1 − *β* err prob = 0.85), and an effect size of 0.15 (*d* = 0.15).The minimum required sample size for correlation analysis was determined to be 392 participants. To account for potential data loss, a design effect of 1.5 was applied, and data were collected from a total of 588 individuals. During data cleaning, individuals who lacked internet access, did not use social media, failed to complete the survey or scale items fully, or reported having diagnosed eating disorder were excluded. After applying the exclusion criteria, analyses were completed with 554 participants (Fig. 1). The study population included non-clinical adults from the general community, rather than patients diagnosed with eating disorders. The inclusion criteria were being between 18 and 64 years of age, living in Kırklareli, having internet access, and using at least one social media platform. The exclusion criteria were: (1) failure to complete all survey items, (2) lack of social media use, (3) lack of internet access, and (4) a self-reported diagnosis of an eating disorder. Participants with incomplete questionnaire or scale data were excluded from the analyses, and all statistical analyses were conducted using complete case data.

This study was conducted in accordance with the principles of the Declaration of Helsinki. Ethical approval was obtained from the Kırklareli University Scientific Research and Publication Ethics Board (approval no. 127460, 11 July 2024). Written informed consent was obtained from all participants prior to data collection.

### Data collection

Data were gathered through face-to-face interviews using structured questionnaires and standardized scales. The questionnaire developed for this cross-sectional study was administered by researchers using the ‘face-to-face interview method’ between 30 July 2024 and 30 August 2024 in the Central District of Kırklareli. The survey included 51 items assessing sociodemographic characteristics, dietary habits, meal patterns, and social media use. In addition, three validated instruments were administered face-to-face:

- The Social Media Impact on Eating Behavior Scale (SESMEB), consisting of 18 items,
- The Digital Healthy Diet Literacy Scale (DHDL), consisting of 4 items, and
- The Bergen Social Media Addiction Scale (BSMAS), consisting of 6 items.

### Assessment of nutritional status

To assess participants’ nutritional status, information was collected on the number of main and snack meals consumed, whether they had received nutrition education, eating-out habits, perceived eating speed, and sleep patterns. These items were included in the “Nutritional Habits” section of the questionnaire.

### Anthropometric measurements

Anthropometric measurements included weight (kg), height (cm), waist circumference (cm), and hip circumference (cm). Body Mass Index (BMI) was calculated as weight (kg) divided by the square of height (m^2^). According to World Health Organization (WHO) standards, BMI < 18.50 kg/m^2^ was classified as “underweight”, 18.50–24.99 kg/m^2^ as “normal”, 25.00–29.99 kg/m^2^ as “pre-obese”, and ≥30.00 kg/m^2^ as “obese” [20].

Waist and hip circumferences were measured by the researchers using standard anthropometric techniques. Waist-to-hip ratio (WHR) values below 0.80 in women were considered low, between 0.80 and 0.84 moderate, and 0.85–1.00 increased health risk. For men, WHR values below 0.90 were low, between 0.90 and 0.94 moderate, and 0.95–1.00 high risk [21].

### Assessment of nutrition and social media use

Participants’ social media habits were evaluated using the “Nutrition and Social Media Use” form, a researcher-developed structured questionnaire, which included questions about the number of social media accounts, duration and daily time of social media use, interest in nutrition/diet topics, following nutrition-related accounts, reasons for following such content, time spent on nutrition-related information, the influence of advertisements on eating habits, consumption of advertised brands, the effect of social media on changing frequently consumed foods, application of diet suggestions from social media, and the impact of food-related posts on appetite and eating behavior.

### Social Media Impact on Eating Behavior Scale (SESMEB)

This scale was selected, because it directly assesses social media’s influence on eating behavior, which is the central focus of the study. With the integration of social media into daily life, the need to examine its effects on eating behavior has emerged. For this purpose, Keser et al. [22] developed the SESMEB in 2020. The 18-item instrument uses a five-point Likert scale ranging from 1 (“Never”) to 5 (“Always”). Total scores range from 18 to 90, with higher scores indicating a greater level of self-reported influence from social media. The internal consistency of the original scale was evaluated using Cronbach’s alpha [22]. In the present study, the Cronbach’s alpha coefficient for SESMEB was calculated as 0.94, indicating excellent reliability. This instrument has demonstrated high internal consistency and construct validity in both its original development study and the Turkish adaptation, confirming its suitability for use in non-clinical adult populations.

### Bergen Social Media Addiction Scale (BSMAS)

The BSMAS was used as a standardized and well-validated measure of social media addiction across multiple cultures.Developed by Andreassen et al., the BSMAS is based on a six-dimensional model of addiction. The Turkish adaptation was conducted by İbrahim Demirci in 2019 [23]. In research and clinical settings, the scale is commonly used to assess the severity of social media addiction-related symptoms rather than to provide a formal diagnosis. It consists of six items rated on a five-point Likert scale ranging from (1) “Very rarely” to (5) “Very often,” with total scores ranging from 6 to 30. Although no strict cutoff exists, obtaining a score of 3 or higher on at least four items—or on all six items for stricter interpretation—suggests a high level of social media addiction. The internal consistency coefficient of the original scale was 0.88 [23]. In this study, the Cronbach’s alpha coefficient for BSMAS was 0.82. This scale has shown strong internal consistency and construct validity across multiple studies, including its Turkish validation, supporting its reliability for evaluating social media addiction tendencies in general adult populations.

### Digital Healthy Diet Literacy Scale—Short Form (DHDL)

The DHDL scale was chosen to quantify participants’ ability to evaluate online nutrition information, representing the construct of digital diet literacy. With the growing use of the Internet for information access, health literacy has become an essential competency, enabling individuals to understand and interpret information necessary for maintaining and improving their health. Low health literacy may lead to poor health decisions and negative outcomes, whereas high literacy supports the comprehension of health information and the adoption of preventive behaviors. The Digital Healthy Diet Literacy Scale (Short Form), originally developed by Duong et al. [24] and adapted into Turkish by Karahan and Eskici [25], consists of four items measuring individuals’ ability to find and evaluate healthy nutrition information online. The scoring index was calculated using the formula Index = (Mean − 1) × 50/3, with possible scores ranging from 0 to 50, where higher scores indicate better literacy [25]. In this study, the Cronbach’s alpha coefficient for DHDL was 0.85. This instrument has demonstrated high internal consistency and satisfactory construct validity in both the original and Turkish versions, confirming its appropriateness for assessing digital diet literacy among community-dwelling adults.

All three standardized scales used in this study are self-report screening instruments and do not substitute for structured clinical diagnostic interviews; therefore, the findings reflect self-reported tendencies related to social media use, digital diet literacy, and social media-linked eating behaviors rather than formal eating-disorder diagnoses.

### Data analysis

The primary outcome variable of the study was social media-related eating behavior, operationalized as total scores on the Social Media Impact on Eating Behavior Scale (SESMEB). The main independent variables were social media addiction (BSMAS scores) and digital healthy diet literacy (DHDL scores). All analyses were conducted on complete cases; participants who did not fully complete the questionnaire or any of the scales were excluded, and no data imputation was performed.

The primary hypotheses were:

1. higher social media addiction (BSMAS) would be positively associated with higher SESMEB scores, and
2. higher digital healthy diet literacy (DHDL) would be associated with more favorable patterns of social media-related eating behavior (lower SESMEB scores).

Descriptive statistics were computed for all variables (mean, standard deviation, frequency, and percentage). The normality of continuous variables was tested using the Shapiro–Wilk test. As SESMEB, BSMAS, and DHDL scores showed non-normal distributions, nonparametric tests were applied. The Mann–Whitney *U* test was used for comparisons between two groups (e.g., gender), and the Kruskal–Wallis test was employed for comparisons among three or more groups (e.g., BMI categories, meal frequency, and categories of social media use). Spearman’s rank correlation coefficients were calculated to examine associations between SESMEB, BSMAS, and DHDL scores. Spearman’s *r* values were interpreted as measures of the strength of association between the main scales.

All analyses were conducted using IBM SPSS Statistics version 26, and statistical significance was set at *p* < 0.05 (two-tailed).

*AI-assisted editing*: The authors used *ChatGPT* (OpenAI; accessed November 2025) for translation and language polishing only; all scientific content is the authors’ own.

## Results

### Participant characteristics

The mean age of participants (*n* = 554) was 31.47 ± 10.45 years; 52.0% were female, 58.5% were single, 43.5% held a university degree, and 46.8% reported that their income equaled expenses. In total, 51.8% had a normal BMI and 72.9% had a normal waist-to-hip ratio, while 21.8% reported regular exercise. The mean duration of social media use was 8.62 ± 3.69 years (Table 1).

**Table 1:** Distribution of participants’ sociodemographic, lifestyle, and health characteristics (*n* = 554)

Variables	*n*	%
*Sex*		
Male	266	48.0
Female	288	52.0
*Age (years) (mean* ± *SD: 31.47* ± *10.45; min: 18, max: 65)*		
<25 years	193	34.8
25–34 years	177	31.9
35–44 years	104	18.8
≥45 years	80	14.4
*Marital status*		
Married	230	41.5
Single	324	58.5
*Education level*		
Primary school	35	6.3
Middle school	38	6.9
High school	229	41.3
University	241	43.5
Master’s/Doctorate	11	2.0
*Income status*		
Income < expenses	209	37.7
Income = expenses	259	46.8
Income > expenses	86	15.5
*Body mass index (BMI)*		
Underweight	30	5.4
Normal	287	51.8
Pre-obese	176	31.8
Obese	61	11.0
*Waist-to-hip ratio*		
Normal	404	72.9
Increased risk	150	27.1
*Regular exercise*		
Yes	121	21.8
No	433	78.2
*Duration of social media use (years) (mean* ± *SD: 8.62* ± *3.69; min: 1, max: 25)*		
≤4 years	73	13.2
5–9 years	203	36.6
≥10 years	278	50.2

### SESMEB scores

SESMEB scores were significantly higher among females, participants under 30 years of age, and single individuals (*p* < 0.05). By BMI category, scores were significantly lower in obese (*p* = 0.032) and pre-obese (*p* = 0.007) groups compared with normal BMI participants. No significant differences were found according to education level, income status, waist-to-hip ratio, or regular exercise (*p* > 0.05) (Table 2).

**Table 2:** Comparison of SESMEB, BSMAS, and DHDL mean scores by participants’ sociodemographic, lifestyle, and health characteristics (*n* = 554)

Variables	*n*	SESMEB Mean ± SD	*p*	BSMAS Mean ± SD	*p*	DHDL Mean ± SD	*p*
*Sex*^a^							
Male	266	33.21 ± 14.50	*p* = 0.024*	13.69 ± 5.51	*p* = 0.055	22.91 ± 13.28	*p* = 0.607
Female	288	34.76 ± 12.94		14.65 ± 5.81		23.50 ± 12.19	
*Age (years)*^a^							
<30 years	287	37.08 ± 13.37	*p* = 0.000***	15.91 ± 5.30	*p* = 0.000***	23.70 ± 11.63	*p* = 0.448
≥30 years	267	30.72 ± 13.35		12.34 ± 5.51		22.70 ± 13.80	
*Marital status*^a^							
Married	230	30.01 ± 12.67	*p* = 0.000***	11.93 ± 5.26	*p* = 0.000***	23.69 ± 14.14	*p* = 0.491
Single	324	36.86 ± 13.75		15.79 ± 5.44		22.89 ± 11.62	
*Education level*^a^							
High school or below	302	34.04 ± 14.11	*p* = 0.753	13.30 ± 5.53	*p* = 0.000***	21.90 ± 13.24	*p* = 0.006**
University or higher	252	33.98 ± 13.27		15.25 ± 5.70		24.80 ± 11.90	
*Income status*^b^							
Income < expenses	209	34.61 ± 14.05		14.43 ± 6.14		21.87 ± 12.29	
Income = expenses	259	32.75 ± 13.27	*p* = 0.086	13.89 ± 5.59	*p* = 0.413	22.74 ± 12.99	*p* = 0.001**
Income > expenses	86	36.38 ± 14.01		14.52 ± 4.78		27.95 ± 11.95	
*BMI*^b^							
Underweight	30	33.83 ± 10.42	*p* = 0.002**	16.50 ± 5.87	*p* = 0.000***	23.47 ± 13.64	*p* = 0.002**
Normal	287	35.60 ± 13.65		14.75 ± 5.62		23.78 ± 12.01	
Pre-obese	176	32.18 ± 13.74		13.86 ± 5.64		24.21 ± 13.71	
Obese	61	31.91 ± 14.74		11.34 ± 5.08		17.62 ± 11.39	
*Waist-to-hip ratio*^a^							
Normal	404	33.53 ± 13.21	*p* = 0.481	14.54 ± 5.59	*p* = 0.008**	23.31 ± 12.30	*p* = 0.849
Increased risk	150	35.31 ± 14.99		13.24 ± 5.86		22.97 ± 13.82	
*History of dieting*^a^							
Yes	159	36.47 ± 13.68	*p* = 0.003**	14.63 ± 5.72	*p* = 0.263	25.13 ± 12.60	*p* = 0.024*
No	395	33.03 ± 13.63		14.01 ± 5.67		22.45 ± 12.70	
*Person determining the diet (n* = *159)*^b^							
Dietitian	83	35.10 ± 13.56		13.73 ± 5.68		23.34 ± 12.81	
Self-directed	60	46.56 ± 13.25	*p* = 0.012*	18.81 ± 5.94	*p* = 0.006**	27.86 ± 11.25	*p* = 0.157
Media-based diet lists	16	35.68 ± 13.04		14.76 ± 5.30		26.87 ± 12.45	
*Regular exercise*^a^							
Yes	121	35.75 ± 15.72	*p* = 0.352	13.68 ± 5.51	*p* = 0.246	25.20 ± 13.20	*p* = 0.046*
No	433	33.53 ± 13.09		14.33 ± 5.73		22.67 ± 12.54	

Bold values indicate statistically significant results

*SESMEB* Effects of Social Media on Eating Behavior, *BSMAS* Bergen Social Media Addiction Scale, *DHDL* Digital Healthy Diet Literacy

* *p* < 0.05; ** *p* < 0.01; *** *p* < 0.001

^a^Mann–Whitney *U* test

^b^Kruskal–Wallis variance analysis were used

### BSMAS scores

BSMAS scores were significantly higher among participants under 30 years, single individuals, and those with university-level or higher education (*p* < 0.05). Obese individuals had significantly lower BSMAS scores than those in the normal (*p* = 0.000), underweight (*p* = 0.000), and pre-obese (*p* = 0.010) groups. Participants with a normal waist-to-hip ratio scored higher than those at risk (*p* = 0.008). No significant associations were observed for sex, income, or exercise status (*p* > 0.05) (Table 2).

### DHDL scores

DHDL scores were significantly higher among participants with university education or higher, those reporting income above expenses, and those exercising regularly (*p* < 0.05). Obese participants scored significantly lower than those in the normal (*p* = 0.002) and pre-obese (*p* = 0.002) groups. No significant differences were detected by sex, age, marital status, or waist-to-hip ratio (*p* > 0.05) (Table 2).

### Associations with dietary habits

SESMEB scores were significantly associated with number of snack meals, receipt of nutrition education, eating outside the home, sleep duration, and eating speed (*p* < 0.05). BSMAS scores differed by number of main and snack meals, eating outside the home, perceived healthy/regular eating, sleep regularity, and eating speed (*p* < 0.05). DHDL scores were higher among those perceiving themselves as eating healthily (*p* = 0.007) and regularly (*p* = 0.004) (Table 3).

**Table 3:** Mean SESMEB, BSMAS, and DHDL scores by dietary habit characteristics (*n* = 554)

Variables	*n*	SESMEB Mean ± SD	*p*	BSMAS Mean ± SD	*p*	DHDL Mean ± SD	*p*
*Number of main meals per day*^b^							
1	18	32.27 ± 15.10	*p* = 0.773	17.44 ± 4.57	*p* = 0.014*	24.07 ± 13.82	*p* = 0.670
2	290	33.93 ± 13.43		14.34 ± 5.82		22.84 ± 12.07	
3	246	34.24 ± 14.00		13.77 ± 5.53		23.61 ± 13.40	
*Number of snack meals per day*^b^							
0	144	30.56 ± 13.12	*p* = 0.000***	13.15 ± 5.51	*p* = 0.019*	22.10 ± 13.85	*p* = 0.664
1	233	35.36 ± 13.32		14.81 ± 5.47		23.64 ± 12.42	
≥2	177	35.05 ± 14.31		14.22 ± 6.02		23.58 ± 12.16	
*Received nutrition education*^a^							
Yes	66	39.77 ± 14.75	*p* = 0.000***	15.45 ± 6.12	*p* = 0.075	25.06 ± 11.31	*p* = 0.195
No	488	33.24 ± 13.40		14.02 ± 5.61		22.97 ± 12.89	
*Eating outside the home*^a^							
Yes	436	34.86 ± 13.76	*p* = 0.001**	14.86 ± 5.61	*p* = 0.000***	23.66 ± 12.58	*p* = 0.122
No	118	30.89 ± 13.16		11.71 ± 5.28		21.61 ± 13.16	
*Do you consider your diet healthy?*^a^							
Yes	246	34.47 ± 14.80	*p* = 0.961	13.44 ± 5.65	*p* = 0.004**	24.83 ± 12.92	*p* = 0.007**
No	308	33.65 ± 12.81		14.79 ± 5.65		21.94 ± 12.43	
*Do you consider your diet regular?*^a^							
Yes	260	33.96 ± 14.17	*p* = 0.584	13.61 ± 5.69	*p* = 0.019*	24.83 ± 13.00	*p* = 0.004**
No	294	34.07 ± 13.33		14.70 ± 5.64		21.79 ± 12.32	
*Sleep irregularity/sleep problems*^a^							
Yes	265	34.06 ± 13.28	*p* = 0.620	14.80 ± 5.92	*p* = 0.034*	22.54 ± 12.43	*p* = 0.218
No	289	33.98 ± 14.14		13.63 ± 5.41		23.84 ± 12.97	
*Eating speed*^b^							
Slow	75	37.17 ± 13.97	*p* = 0.002**	15.53 ± 5.68	*p* = 0.037*	22.61 ± 12.65	*p* = 0.531
Moderate	276	32.71 ± 13.10		13.82 ± 5.84		23.55 ± 12.36	
Fast	166	33.25 ± 13.47		13.87 ± 5.17		23.54 ± 12.91	
Very fast	37	40.83 ± 16.23		15.67 ± 6.30		20.60 ± 14.69	

Bold values indicate statistically significant results

*SESMEB* Effects of Social Media on Eating Behavior, *BSMAS* Bergen Social Media Addiction Scale, *DHDL* Digital Healthy Diet Literacy

* *p* < 0.05; ** *p* < 0.01; *** *p* < 0.001

^a^Mann–Whitney *U* test

^b^Kruskal–Wallis variance analysis were used

### Associations with social media use

SESMEB scores were significantly related to number of social media accounts, duration of use, perceived importance of “likes,” participation in nutrition-related groups, influence of advertisements, and time spent seeking nutrition information (*p* < 0.05). Except for “reason for following nutrition-related information,” BSMAS and DHDL scores significantly differed across all social media use variables (*p* < 0.05) (Table 4).

**Table 4:** Mean SESMEB, BSMAS, and DHDL scores by social media use characteristics (*n* = 554)

Variables	*n*	SESMEB Mean ± SD	*p*	BSMAS Mean ± SD	*p*	DHDL Mean ± SD	*p*
*Number of social media accounts*^a^							
<3	323	31.93 ± 13.20	*p* = 0.000***	12.97 ± 5.62	*p* = 0.000***	21.54 ± 13.00	*p* = 0.000***
≥3	231	36.93 ± 13.93		15.89 ± 5.35		25.57 ± 11.95	
*Duration of social media use*^a^							
<10 years	276	34.27 ± 14.18	*p* = 0.843	13.56 ± 5.74	*p* = 0.007**	21.15 ± 12.21	*p* = 0.000***
≥10 years	278	33.76 ± 13.27		14.82 ± 5.58		25.28 ± 12.90	
*Daily time spent on social media*^a^							
<3 h	297	30.81 ± 13.53	*p* = 0.000***	12.15 ± 5.11	*p* = 0.000***	23.21 ± 13.35	*p* = 0.949
≥3 h	257	37.71 ± 13.02		16.55 ± 5.41		23.23 ± 11.97	
*Attaching importance to the number of “likes”*^a^							
Yes	97	39.39 ± 13.35	*p* = 0.000***	16.85 ± 4.90	*p* = 0.000***	27.31 ± 11.25	*p* = 0.001**
No	457	32.87 ± 13.54		13.62 ± 5.69		22.35 ± 12.86	
*Interest in nutrition/diet topics*^a^							
Yes	283	38.14 ± 13.53	*p* = 0.000***	14.75 ± 5.24	*p* = 0.004**	25.58 ± 11.30	*p* = 0.000***
No	271	29.71 ± 12.56		13.60 ± 6.08		20.75 ± 13.64	
*Membership in nutrition-related social media groups*^a^							
Yes	67	43.19 ± 15.08	*p* = 0.000***	15.86 ± 4.70	*p* = 0.003**	29.22 ± 11.01	*p* = 0.000***
No	487	32.75 ± 13.04		13.96 ± 5.78		22.39 ± 12.73	
*Following accounts with useful nutrition information daily*^a^							
Yes	185	39.86 ± 12.79	*p* = 0.000***	15.58 ± 5.07	*p* = 0.000***	27.22 ± 9.89	*p* = 0.000***
No	369	31.08 ± 13.24		13.49 ± 5.86		21.21 ± 13.49	
*Reason for following nutrition-related information*^b^							
To protect/improve health	166	35.10 ± 13.54	*p* = 0.000***	14.57 ± 5.62	*p* = 0.149	22.69 ± 12.60	*p* = 0.418
Weight control/weight loss	86	39.53 ± 14.66		14.75 ± 5.63		21.80 ± 11.33	
Disease-related nutrition information	46	35.26 ± 12.39		12.58 ± 4.88		24.27 ± 11.59	
To obtain up-to-date information	256	31.23 ± 13.11		14.04 ± 5.86		23.86 ± 13.42	
*Time spent seeking nutrition information on social media*^a^							
None	340	30.52 ± 12.83	*p* = 0.000***	13.51 ± 5.91	*p* = 0.000***	21.29 ± 13.50	*p* = 0.000***
≥1 h	214	39.57 ± 13.28		15.26 ± 5.15		26.28 ± 10.71	
*Use of apps/programs to control social media use*^a^							
Yes	61	42.29 ± 14.10	*p* = 0.000***	16.52 ± 5.33	*p* = 0.000***	26.63 ± 11.51	*p* = 0.044*
No	493	32.99 ± 13.33		13.90 ± 5.67		22.80 ± 12.81	
*Perceiving that social media ads/promotions affect eating habits*^a^							
Yes	211	41.86 ± 13.29	*p* = 0.000***	15.82 ± 5.25	*p* = 0.000***	26.10 ± 11.14	*p* = 0.000***
No	343	29.19 ± 11.60		13.18 ± 5.72		21.45 ± 13.31	
*Consuming more of a brand’s products after exposure to its ads on social media*^a^							
Yes	228	40.08 ± 13.91	*p* = 0.000***	15.63 ± 5.58	*p* = 0.000***	25.42 ± 12.32	*p* = 0.001**
No	326	29.77 ± 11.88		13.18 ± 5.55		21.68 ± 12.79	
*Social media influencing changes in routinely consumed foods*^a^							
Yes	138	44.21 ± 14.65	*p* = 0.000***	15.89 ± 5.73	*p* = 0.000***	27.11 ± 11.37	*p* = 0.000***
No	416	30.63 ± 11.57		13.62 ± 5.57		21.93 ± 12.89	
*Applying nutrition/diet recommendations followed on social media*^a^							
Yes	153	41.84 ± 13.59	*p* = 0.000***	15.83 ± 5.52	*p* = 0.000***	27.31 ± 10.19	*p* = 0.000***
No	401	31.03 ± 12.56		13.56 ± 5.63		21.66 ± 13.24	
*Trying a food product due to social media influence*^a^							
Yes	265	40.40 ± 12.96	*p* = 0.000***	15.56 ± 5.55	*p* = 0.000***	25.25 ± 11.20	*p* = 0.000***
No	289	28.16 ± 11.65		12.93 ± 5.52		21.36 ± 13.73	

Bold values indicate statistically significant results

*SESMEB* Effects of Social Media on Eating Behavior, *BSMAS* Bergen Social Media Addiction Scale, *DHDL* Digital Healthy Diet Literacy

* *p* < 0.05; ** *p* < 0.01; *** *p* < 0.001

^a^Mann–Whitney *U* test

^b^Kruskal–Wallis variance analysis were used

### Inter-scale correlations

Positive and statistically significant correlations were observed between the scales. SESMEB was moderately correlated with BSMAS (*r* = 0.443, *p* < 0.001) and weakly correlated with DHDL (*r* = 0.182, *p* < 0.001). A weak but statistically significant correlation was also found between BSMAS and DHDL (*r* = 0.131, *p* = 0.002) (Table 5).

**Table 5:** Correlations among SESMEB, BSMAS, and DHDL (*n* = 554)

	SESMEB	BSMAS	DHDL
SESMEB	1.00	*r* = 0.443 *p* = 0.000**	*r* = 0.182 *p* = 0.000**
BSMAS	–	1.00	*r* = 0.131 *p* = 0.002**
DHDL	–	–	1.00

Bold values indicate statistically significant correlations

*SESMEB* Effects of Social Media on Eating Behavior, *BSMAS* Bergen Social Media Addiction Scale, *DHDL* Digital Healthy Diet Literacy

** *p* < 0.01. Spearman correlation test

## Discussion

Social media platforms are among the primary means of communication worldwide and have become integral to daily life by accelerating access to information [26]. The increasing use of these platforms can have both beneficial and adverse effects on health and nutritional behaviors. In this study, the relationships among social media addiction (BSMAS), digital healthy diet literacy (DHDL), and social media-related eating behaviors (SESMEB) in a community sample of adults.

Globally, social media use was reported to have reached 49% of the world’s population in 2020, with platforms such as Facebook, YouTube, Snapchat, Instagram, WeChat, and TikTok creating new digital environments particularly for young users [27]. In Türkiye, the average daily time spent on social media has been reported as 2 h and 44 min [28]. Our findings underscore the widespread nature of social media use: 32.1% of participants had two social media accounts, mean duration of use was approximately 8 years, and 22% reported spending more than 3 h/day on social media—figures broadly consistent with national estimates.

Evaluation of SESMEB, BSMAS, and DHDL scores across sex, age, marital status, education, income, and health parameters suggests that the impact of social media use on eating behavior is multidimensional. Higher scores among women and participants under 30 indicate that social media may be more closely associated with eating behaviors in younger and female users. This aligns with existing evidence showing that social media exposure contributes to body image concerns, internalization of ideal body types, and restrictive or compensatory behaviors that increase vulnerability to disordered eating [29–33]. Previous studies have also reported associations between social media addiction, impaired body image, and disordered eating both directly and indirectly [30, 31, 34].

With respect to BMI, SESMEB, BSMAS, and DHDL scores were significantly lower among obese participants compared to those with normal BMI, indicating that individuals with higher body weight may experience weaker associations between social media use and eating-related behaviors. Similarly, participants with an at-risk waist-to-hip ratio showed higher BSMAS scores than those with a normal ratio, suggesting that body composition may influence patterns of social media engagement. These findings may reflect differential internalization of body ideals across body size groups rather than a direct effect of social media exposure. Prior research among Israeli university students reported that social media use was positively correlated with disordered eating symptoms, with body dissatisfaction mediating this association [35], This pattern is consistent with previous studies linking higher social media addiction, lower body satisfaction, and greater vulnerability to disordered eating [29, 34].

In terms of dietary habits, SESMEB scores were associated with number of snack meals, receipt of nutrition education, eating outside the home, sleep duration, and eating speed; BSMAS scores were associated with number of main and snack meals, eating outside the home, perceived healthy/regular eating, sleep regularity, and eating speed. These findings suggest that social media engagement may co-occur with irregular eating patterns and lifestyle behaviors, such as disrupted sleep or inconsistent meal timing [36]. Higher DHDL scores among participants with higher education and income, together with its association with perceived healthy eating, support the notion that the ability to critically evaluate online nutrition information contributes to healthier dietary patterns. Similar trends have been reported among nursing and medical students during the pandemic [17], as well as in international studies linking greater digital health literacy with healthier dietary [30, 32, 37, 38].

Peer interactions on social media can also shape nutritional attitudes and behaviors. For example, Thaichon and Quach demonstrated that the prevalence of fast-food advertising on social media significantly affects adolescents’ attitudes, purchase behaviors, and overall dietary habits [39]. Consistent with this, a considerable proportion of participants in this study reported following nutrition-related content, with more than one-third spending dedicated time on diet-related information. While this demonstrates social media’s potential for health promotion, the accuracy of such content remains variable and requires critical evaluation.

Both positive and negative impacts of social media on eating behaviors were observed. While 41.2% of participants reported applying nutrition advice obtained from social media and 47.8% tried new food products due to social media exposure, the prevalence of high-calorie, low-nutrient food imagery on these platforms—reported to comprise 68% of shared food visuals, with only 22% depicting fruits and vegetables—may trigger unhealthy behaviors, particularly among youth [40]. Although social media can foster awareness via engaging content and communities (e.g., campaigns increasing help-seeking among men for eating-disorder concerns [41] and initiatives enhancing the visual appeal of healthy foods and disseminating key dietary information [42]), visually enticing presentations of unhealthy foods may prompt impulsive eating and irregular patterns. In Türkiye, adults with negative eating attitudes were also found to follow nutrition content more closely on social media [43], suggesting that the motivation to seek diet-related information may coexist with maladaptive tendencies. These findings are consistent with cognitive–behavioral and affect-regulation models of disordered eating, in which social comparison and emotional distress contribute to irregular or compulsive eating behaviors [44].

Significant correlations among SESMEB, BSMAS, and DHDL scores further indicate that social media addiction, digital diet literacy, and eating behaviors are inter-related. While social media may enhance awareness of healthy nutrition, it also increases exposure to misinformation. Strengthening digital diet literacy could, therefore, help mitigate potential adverse influences by fostering critical appraisal and evidence-based dietary decision-making. Accordingly, enhancing digital diet literacy is critical to support critical appraisal of online content and promote healthy eating—conclusions in line with prior literature [17, 30, 32, 37, 38].

This study is among the few conducted in Türkiye to investigate relationships among social media addiction, digital healthy diet literacy, and eating behaviors in adults. The findings demonstrate that social media may influence dietary attitudes both positively and negatively. The positive association between social media addiction and digital healthy diet literacy suggests a dual nature: on one hand, addiction may exacerbate unhealthy eating; on the other, accurate information accessed via these platforms may enhance digital diet literacy.

Lower levels of both social media addiction and digital diet literacy among obese individuals imply that the effects of social media vary by individual, psychological, and lifestyle factors. Overall, social media presents both risks and opportunities in shaping eating behaviors. We recommend the development of social media-based, reliable, and evidence-based content to raise awareness of healthy eating, protect against misinformation, and strengthen digital health literacy.

Recent studies have further illuminated the psychosocial and behavioral pathways through which digital environments shape eating patterns. In a study of women of reproductive age, higher social media use was associated with greater emotional eating, suggesting that digital appearance pressures and online comparison may act as triggers for dysregulated eating [45]. Similarly, research among esports players (*n* = 248) identified a co-occurrence of night eating syndrome and food addiction, linking these to extended screen exposure and irregular meal timing [46]. Moreover, emerging evidence highlights the role of nutrition misinformation—often amplified by social media algorithms and AI-generated content—in promoting guilt, restrictive behaviors, and distorted body-related cognitions [47]. Collectively, these findings emphasize that eating behaviors do not occur in isolation but are shaped by technological, cognitive, and cultural influences within the digital ecosystem.

Future research should include longitudinal designs encompassing diverse age and socioeconomic groups and multiple social media platforms.

## Strengths and limitations of the study

Strengths of this study include its relatively large sample size; the simultaneous assessment of social media addiction, digital diet literacy, and eating behaviors using three validated instruments (SESMEB, BSMAS, and DHDL); and the inclusion of sociodemographic, lifestyle, and anthropometric variables that allowed for a multidimensional analysis.

Nevertheless, several limitations should be noted. First, the cross-sectional design precludes causal inference, and the observed associations should be interpreted as correlational. Second, all measures were based on self-report data, which may be subject to recall bias, under-reporting, and social desirability effects. Moreover, the instruments used (SESMEB, BSMAS, and DHDL) are self-report screening scales and should not be regarded as substitutes for structured clinical diagnostic interviews. Third, although individuals with diagnosed eating disorders were excluded, structured clinical interviews were not conducted to verify participants’ mental health status. Fourth, the study did not separately analyze the distinct effects of specific social media platforms, which may differ in their visual and interactive characteristics. Fifth, the sample was restricted to adults residing in Kırklareli, Türkiye, and primarily represented a non-clinical community population, which may limit generalizability to other regions, age groups, or clinical populations.

Future research should employ longitudinal or experimental designs, include more diverse and representative samples, and incorporate objective behavioral or clinical measures to further elucidate the mechanisms linking social media engagement, digital literacy, and eating behaviors.

## Data Availability

The data that support the findings of this study are available from the corresponding author upon reasonable request.
